# The genome sequence of the Brown Argus, *Aricia agestis *(Denis & Schiffermüller, 1775)

**DOI:** 10.12688/wellcomeopenres.19784.1

**Published:** 2023-08-10

**Authors:** Alex Hayward, Konrad Lohse, Roger Vila, Dominik R. Laetsch, Johanna S.U. Hedlund, Wellcome Sanger Institute Tree of Life programme, Wellcome Sanger Institute Scientific Operations: DNA Pipelines collective, Tree of Life Core Informatics collective, Darwin Tree of Life Consortium

**Affiliations:** 1 College of Life and Environmental Sciences, University of Exeter, Exeter, England, UK; 2 Institute of Ecology and Evolution, The University of Edinburgh, Edinburgh, Scotland, UK; 3 Institut de Biologia Evolutiva, CSIC - Universitat Pompeu Fabra, Barcelona, Spain; 4 Department of Evolutionary Ecology, Lund University, Lund, Sweden

**Keywords:** Aricia agestis, Brown Argus, genome sequence, chromosomal, Lepidoptera

## Abstract

We present genome assemblies from two male *Aricia agestis specimens* (the Brown Argus; Arthropoda; Insecta; Lepidoptera; Lycaenidae). The genome sequences are 435.3 and 437.4 megabases in span. Each assembly is scaffolded into 23 chromosomal pseudomolecules, including the Z sex chromosome. The mitochondrial genomes were assembled and are 15.47 and 15.45 kilobases in length. Gene annotation of these assemblies on Ensembl identified 12,688 and 12,654 protein coding genes.

## Species taxonomy

Eukaryota; Metazoa; Eumetazoa; Bilateria; Protostomia; Ecdysozoa; Panarthropoda; Arthropoda; Mandibulata; Pancrustacea; Hexapoda; Insecta; Dicondylia; Pterygota; Neoptera; Endopterygota; Amphiesmenoptera; Lepidoptera; Glossata; Neolepidoptera; Heteroneura; Ditrysia; Obtectomera; Papilionoidea; Lycaenidae; Polyommatinae; *Aricia*; *Aricia agestis* (Denis & Schiffermüller, 1775) (NCBI:txid91739).

## Background

The Brown Argus ( *Aricia agestis*) is a small butterfly, 24–32 mm in wingspan ( Aagaard *et al.*, 2002). As its name implies, the upper wings of both the male and the female are brown, and orange spots run along all four white wing edges. These resemble the many eyes of Argus, the giant from Greek mythology, which provides the inspiration for its common name. The Brown Argus is a member of the ‘blues’ or Polyommatinae subfamily of Lycaenidae, so named because their furry bodies resemble that of wolves, but the species is not sexually dimorphic, unlike many of the blues. The Brown Argus is very similar in appearance to the Northern Brown Argus ( *Aricia artaxerxes*), which in the UK occurs only in Scotland and northern England and has distinctive white spots on the upper forewing. F _1_ and F _2_ hybrids of these two species can be produced in the lab ( Jarvis, 1959). *A. montensis* and *A. cramera*, also related and hardly distinguishable by external morphology, occur in the Iberian Peninsula, North Africa and, in the case of the latter, in the Balearic Islands and Sardinia. *Aricia agestis* has a parapatric distribution with all these taxa, and hybrids seem to occur at the contact zones ( Sañudo-Restrepo *et al.*, 2013; Vodă *et al.*, 2015).

The range of the Brown Argus centres around the western Palaearctic, reaching southern Scandinavia in the north and Sicily and Crete in the south, but extends as far as Amur and Siberia in the eastern Palaearctic ( Tolman & Lewington, 2008). The preferred habitat of the Brown Argus is flowery grasslands on calcareous soil, where its eggs are laid on the upper surface of leaves from Common Rock-rose ( *Helianthemum nummularium*) or certain Geraniaceae species ( Tolman & Lewington, 2008). A northward range expansion has been demonstrated in the UK, where populations at the range limit appear to have switched mostly to Geraniaceae host plants ( Bridle *et al.*, 2014; Buckley *et al.*, 2012; de Jong *et al.*, 2022; Pateman *et al.*, 2012). Within the southern part of its range, the Brown Argus produces several generations per year, and is possibly trivoltine in the Mediterranean, whereas it is bivoltine or univoltine, at higher latitudes ( Bury, 2016).

The caterpillar of the Brown Argus feeds on the host plant where it hatches, with early larval stages cutting distinctive window-shaped holes in the lower leaf surface, while later stages consume the entire leaf blade. The caterpillar is also attended to by ants, most often by *Lasius* spp. or *Myrmica sabuleti* ( Bury, 2016; Tolman & Lewington, 2008). Symbiosis between ants and lycaenid butterflies is common, and can be obligate and species-specific, or facultative. In the Brown Argus it appears to be the latter ( Fiedler & Hummel, 1995). It diapauses as larvae during winter, and then pupate the subsequent spring ( Tolman & Lewington, 2008). Pupae are brought underground by ants, but the degree of symbiosis remains unclear ( Bury, 2016). The Brown Argus is a common butterfly where it occurs, and is not regarded as under threat by the IUCN.

The number of chromosome pairs in *Aricia agestis* has been recorded as 23 ( Federley, 1938; Robinson, 1971) and 24 ( Lorković, 1941); but note that some of these studies may refer to the closely related *A. artaxerxes*, especially Federley (1938). The genome of the Brown Argus will be useful for further studies on host plant shifts associated with climate change, as well as ecological specialisation and speciation in the genus *Aricia*.

## Genome sequence report

**Figure 1.**
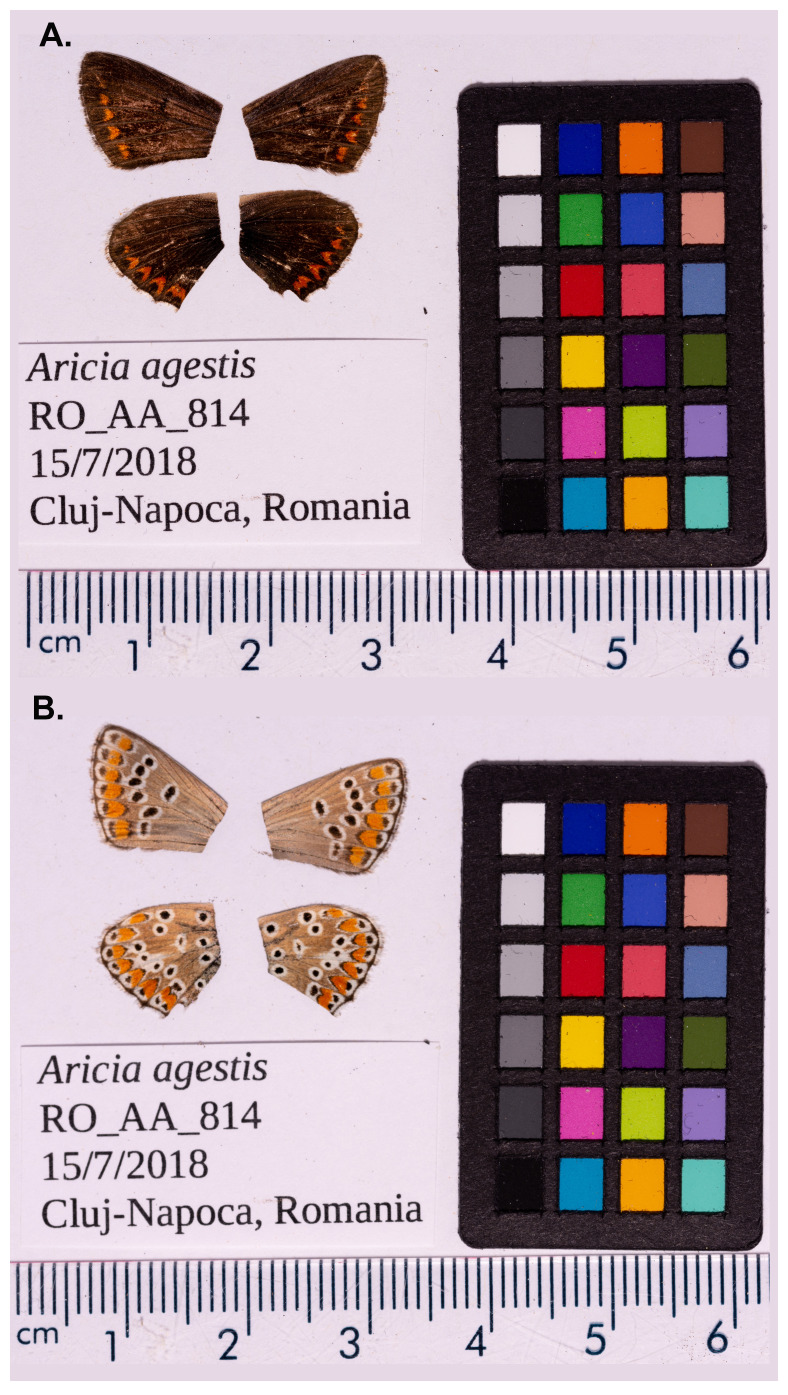
Photograph of an *Aricia agestis* (ilAriAges1) specimen used for genome sequencing. ( **A**) Dorsal surface view of wings ( **B**) Ventral surface view of wings.

The genome was sequenced from two male *Aricia agestis* specimens ( Figure 1) collected from Romania (46.83, 23.63).

For the assembly ilAriAges1.1, a total of 54-fold coverage in Pacific Biosciences single-molecule HiFi long reads and 85-fold coverage in 10X Genomics read clouds was generated. Primary assembly contigs were scaffolded with chromosome conformation Hi-C data. Manual assembly curation corrected two missing joins or misjoins. The final ilAriAges1.1 assembly has a total length of 435.3 Mb in 25 sequence scaffolds with a scaffold N50 of 19.0 Mb ( Table 1).

**Table 1.** Genome data for *Aricia agestis*, ilAriAges1.1 and ilAriAges2.1.

Project accession data		
Assembly identifiers	ilAriAges1.1 and ilAriAges2.1	
Species	*Aricia agestis*	
Specimen	ilAriAges1 and ilAriAges2	
NCBI taxonomy ID	91739	
BioProject	PRJEB42115	
BioSample ID	SAMEA7523300	
Isolate information	ilAriAges1, male: whole organism (DNA sequencing and Hi-C scaffolding) ilAriAges2, male: whole organism (DNA sequencing and Hi-C scaffolding)	
Assembly metrics *	ilAriAges1.1	ilAriAges2.1
Consensus quality (QV) ( *≥ 50*)	56.1	56.4
*k*-mer completeness ( *≥ 95%*)	99.99%	99.99%
BUSCO ** ( *C ≥ 95%*)	C:97.3%[S:97.0%,D:0.3%], F:0.6%,M:2.1%,n:5,286	C:97.2%[S:96.8%,D:0.4%], F:0.5%,M:2.3%,n:5,286
Percentage of assembly mapped to chromosomes ( *≥ 95%*)	99.98%	99.96%
Sex chromosomes ( *localised homologous pairs*)	Z chromosome	Z chromosome
Organelles ( *complete single alleles*)	Mitochondrial genomes assembled	
Raw data accessions	ilAriAges1	ilAriAges2
PacificBiosciences SEQUEL II	ERR6544653, ERR9763977	ERR6544653, ERR9763977
10X Genomics Illumina	ERR6363252, ERR9580457, ERR6363251, ERR9580455	ERR6363251, ERR9580455, ERR9580456, ERR9580458
Hi-C Illumina	ERR6363256, ERR9580459, ERR9580461, ERR6363254	ERR6363254, ERR6363255, ERR6363257, ERR9580460
Genome assembly	ilAriAges1.1	ilAriAges2.1
Assembly accession	GCA_905147365.1	GCA_944452695.1
*Accession of alternate haplotype*	GCA_905147165.1	GCA_944452645.1
Span (Mb)	435.3	437.4
Number of contigs	30	50
Contig N50 length (Mb)	17.8	15.3
Number of scaffolds	25	29
Scaffold N50 length (Mb)	19.0	19.1
Longest scaffold (Mb)	42.2	42.5
Genome annotation	ilAriAges1.1	ilAriAges2.1
Number of protein-coding genes	12,688	12,654
Number of non-coding genes	2,174	2,271
Number of gene transcripts	24,237	24,902

* Assembly metric benchmarks are adapted from column VGP-2020 of “Table 1: Proposed standards and metrics for defining genome assembly quality” from ( Rhie *et al.*, 2021). ** BUSCO scores based on the lepidoptera_odb10 BUSCO set using v5.3.2. C = complete [S = single copy, D = duplicated], F = fragmented, M = missing, n = number of orthologues in comparison. Full sets of BUSCO scores are available for ilAriAges1.1 and ilAriAges2.1.

For the assembly ilAriAges2.1, a total of 56-fold coverage in Pacific Biosciences single-molecule HiFi long reads and 80-fold coverage in 10X Genomics read clouds was generated. Manual assembly curation corrected 15 missing joins or misjoins and removed six haplotypic duplications, reducing the scaffold number by 27.5%, and increasing the scaffold N50 by 0.71%. The final ilAriAges2.1 assembly has a total length of 437.4 Mb in 29 sequence scaffolds with a scaffold N50 of 19.1 Mb ( Table 1).

**Figure 2.**
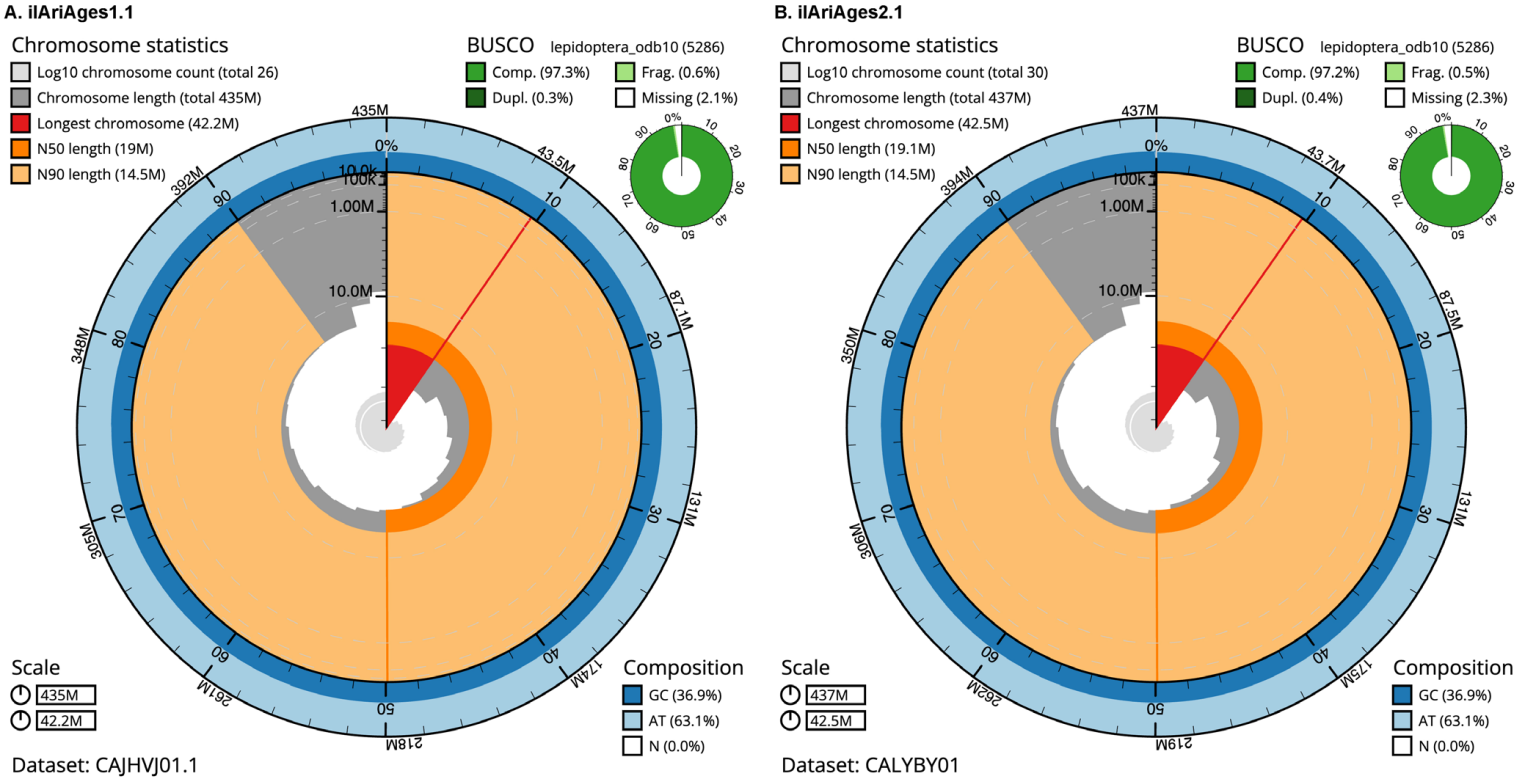
Genome assembly of *Aricia agestis*: metrics. The BlobToolKit Snailplot shows N50 metrics and BUSCO gene completeness. The main plot is divided into 1,000 size-ordered bins around the circumference with each bin representing 0.1% of the assembly. The distribution of sequence lengths is shown in dark grey with the plot radius scaled to the longest sequence present in the assembly Orange and pale-orange arcs show the N50 and N90 sequence lengths respectively. The pale grey spiral shows the cumulative sequence count on a log scale with white scale lines showing successive orders of magnitude. The blue and pale-blue area around the outside of the plot shows the distribution of GC, AT and N percentages in the same bins as the inner plot. A summary of complete, fragmented, duplicated and missing BUSCO genes in the lepidoptera_odb10 set is shown in the top right for each figure. Interactive versions of these figures are available for ilAriAges1.1 and ilAriAges2.1.

For both assemblies, most of the assembly sequence was assigned to 23 chromosomal-level scaffolds, representing 22 autosomes and the Z sex chromosome. Chromosome-scale scaffolds confirmed by the Hi-C data are named in order of size ( Figure 2– Figure 5; Table 2 and Table 3). While not fully phased, each deposited assembly is of one haplotype. Contigs corresponding to the second haplotype have also been deposited. The mitochondrial genome for each specimen was also assembled and can be found as a contig within the multifasta file of the genome submission.

**Figure 3.**
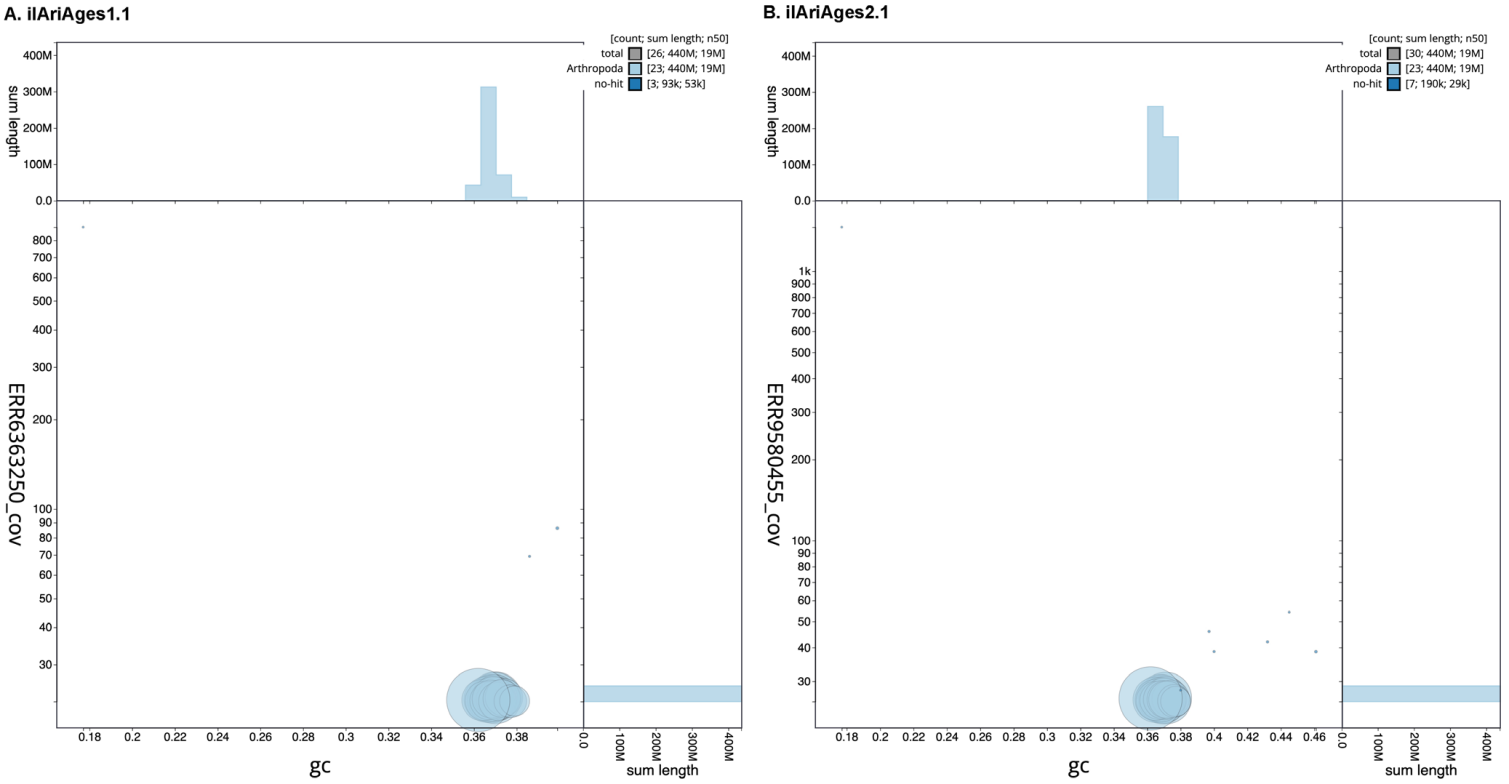
Genome assembly of *Aricia agestis*: BlobToolKit GC-coverage plot. Scaffolds are coloured by phylum. Circles are sized in proportion to scaffold length. Histograms show the distribution of scaffold length sum along each axis. Interactive versions of these figures are available for ilAriAges1.1 and ilAriAges2.1.

**Figure 4.**
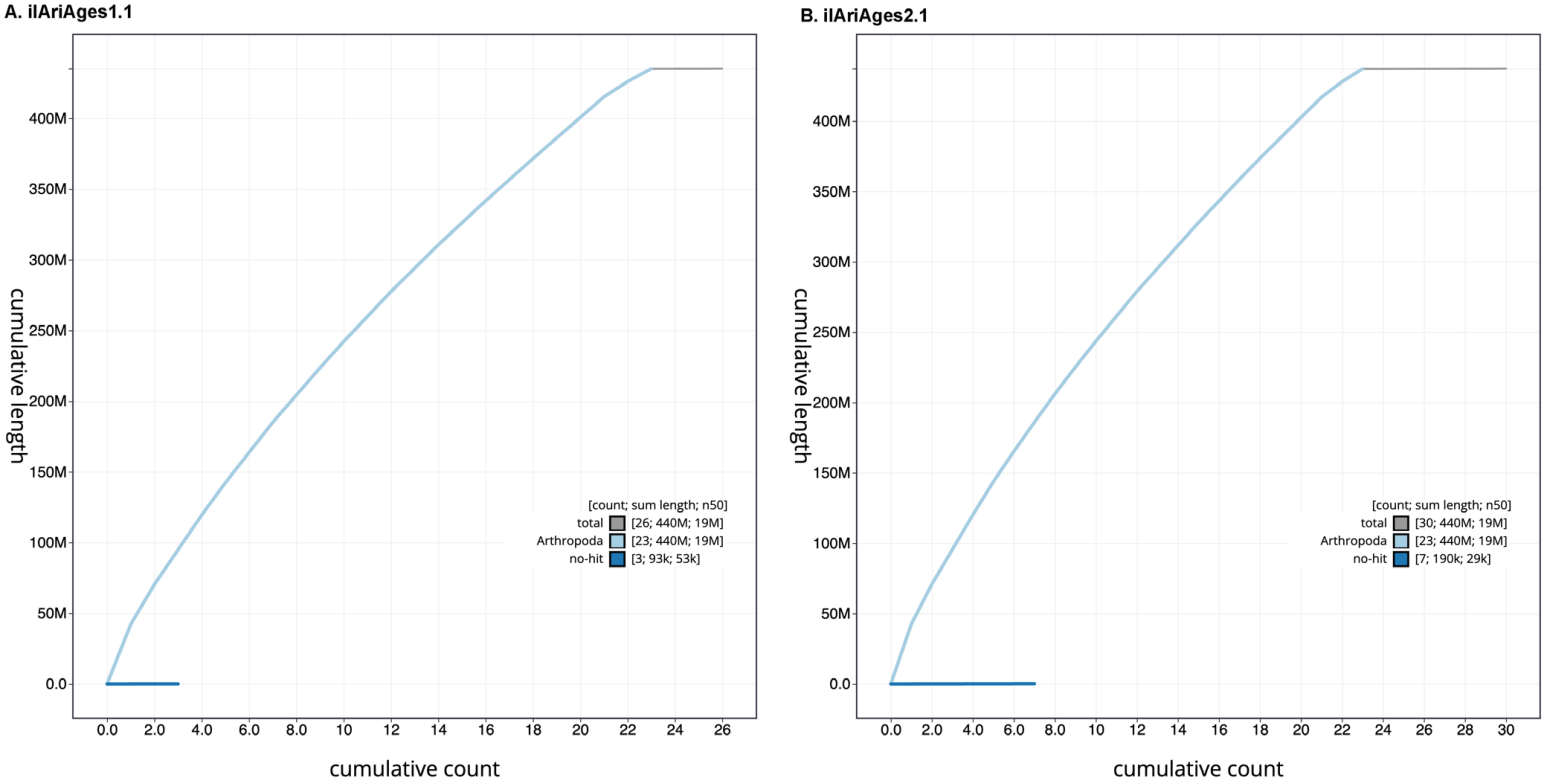
Genome assembly of *Aricia agestis*: BlobToolKit cumulative sequence plot. The grey line shows cumulative length for all scaffolds. Coloured lines show cumulative lengths of scaffolds assigned to each phylum using the buscogenes taxrule. Interactive versions of these figures are available for ilAriAges1.1 and ilAriAges2.1.

**Figure 5.**
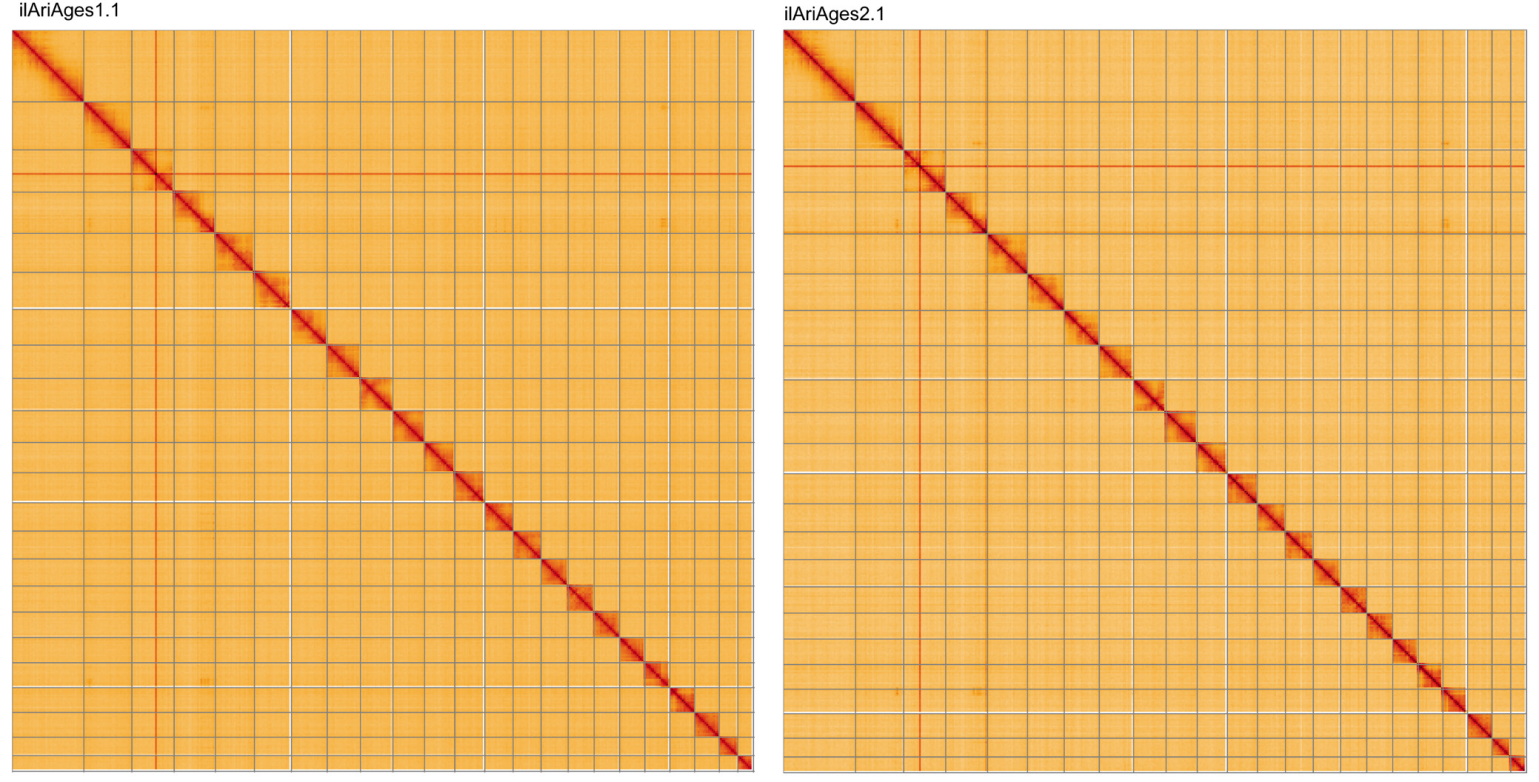
Genome assembly of *Aricia agestis* Hi-C contact map of the ilAriAges1.1 and ilAriAges2.1 assemblies, visualised using HiGlass. Chromosomes are shown in order of size from left to right and top to bottom. Interactive versions of these figures may be viewed for ilAriAges1.1 and ilAriAges2.1.

**Table 2.** Chromosomal pseudomolecules in the genome assembly of *Aricia agestis*, ilAriAges1.

INSDC accession	Chromosome	Length (Mb)	GC%
LR990257.1	1	28.2	37.0
LR990258.1	2	24.84	37.0
LR990259.1	3	24.27	37.0
LR990260.1	4	22.86	37.0
LR990261.1	5	21.7	36.5
LR990262.1	6	21.0	37.0
LR990263.1	7	19.55	36.5
LR990264.1	8	19.03	37.0
LR990265.1	9	18.58	37.0
LR990266.1	10	17.77	36.5
LR990267.1	11	17.6	37.0
LR990268.1	12	16.64	37.0
LR990269.1	13	16.39	37.0
LR990270.1	14	15.88	36.5
LR990271.1	15	15.27	37.0
LR990272.1	16	15.02	36.5
LR990273.1	17	14.82	37.0
LR990274.1	18	14.66	37.0
LR990275.1	19	14.51	37.5
LR990276.1	20	14.5	37.5
LR990277.1	21	10.87	37.5
LR990278.1	22	9.09	38.0
LR990256.1	Z	42.15	36.0
LR990279.1	MT	0.02	18.0

**Table 3.** Chromosomal pseudomolecules in the genome assembly of *Aricia agestis*, ilAriAges2.

INSDC accession	Chromosome	Length (Mb)	GC%
OX101760.1	1	28.33	37.0
OX101761.1	2	24.72	37.0
OX101762.1	3	24.63	37.0
OX101763.1	4	23.56	37.0
OX101764.1	5	21.53	36.5
OX101765.1	6	20.64	37.0
OX101766.1	7	20.17	37.0
OX101767.1	8	19.13	37.0
OX101768.1	9	18.35	37.0
OX101769.1	10	17.71	36.5
OX101770.1	11	17.7	37.0
OX101771.1	12	16.44	37.0
OX101772.1	13	16.41	37.0
OX101773.1	14	15.98	36.5
OX101774.1	15	15.46	37.0
OX101775.1	16	15.28	36.5
OX101776.1	17	15.03	37.0
OX101777.1	18	14.49	37.5
OX101778.1	19	14.48	37.0
OX101779.1	20	14.38	37.0
OX101780.1	21	11.03	37.5
OX101781.1	22	9.22	37.5
OX101759.1	Z	42.5	36.0
OX101782.1	MT	0.02	18.0

The estimated Quality Value (QV) of the final ilAriAges1.1 assembly is 56.1 with *k*-mer completeness of 99.99%, and the assembly has a BUSCO v5.3.2 completeness of 97.3% (single = 97.0%, duplicated = 0.3%), using the lepidoptera_odb10 reference set ( *n* = 5,286).

The estimated Quality Value (QV) of the final ilAriAges2.1 assembly is 56.4 with *k*-mer completeness of 99.99%, and the assembly has a BUSCO v5.3.2 completeness of 97.2% (single = 96.8%, duplicated = 0.4%), using the lepidoptera_odb10 reference set ( *n* = 5,286).

Metadata for specimens, spectral estimates, sequencing runs, contaminants and pre-curation assembly statistics can be found at https://links.tol.sanger.ac.uk/species/91739.

## Genome annotation report

The *Aricia agestis* genome assembly (ilAriAges1.1, GCA_905147365.1) was annotated using the Ensembl rapid annotation pipeline ( Table 1; https://rapid.ensembl.org/Aricia_agestis_GCA_905147365.1/Info/Index). The resulting annotation includes 24,237 transcribed mRNAs from 12,688 protein-coding and 2,174 non-coding genes.

The *Aricia agestis* genome assembly (ilAriAges2.1, GCA_944452695.1) was annotated using the Ensembl rapid annotation pipeline ( Table 1; https://rapid.ensembl.org/Aricia_agestis_GCA_944452695.1/Info/Index). The resulting annotation includes 24,902 transcribed mRNAs from 12,654 protein-coding and 2,271 non-coding genes.

## Methods

### Sample acquisition and nucleic acid extraction

Two male *Aricia agestis* (ToLIDs ilAriAges1 and ilAriAges2) were collected from Cluj-Napoca, Romania (latitude 46.83, longitude 23.63) on 2018-07-15 during the daytime using a handnet. The collectors were Konrad Lohse (University of Edinburgh), Alex Hayward (University of Exeter), Dominik Laetsch (University of Edinburgh), and Roger Vila (Institut de Biologia Evolutiva). The specimen was identified by Roger Vila and then snap-frozen from live in a dry shipper.

DNA was extracted at the Tree of Life laboratory, Wellcome Sanger Institute (WSI). The ilAriAges1 sample was weighed and dissected on dry ice with tissue set aside for Hi-C sequencing. Tissue from the whole organism tissue was disrupted using a Nippi Powermasher fitted with a BioMasher pestle. High molecular weight (HMW) DNA was extracted using the Qiagen MagAttract HMW DNA extraction kit. Low molecular weight DNA was removed from a 20 ng aliquot of extracted DNA using the 0.8X AMpure XP purification kit prior to 10X Chromium sequencing; a minimum of 50 ng DNA was submitted for 10X sequencing. HMW DNA was sheared into an average fragment size of 12–20 kb in a Megaruptor 3 system with speed setting 30. Sheared DNA was purified by solid-phase reversible immobilisation using AMPure PB beads with a 1.8X ratio of beads to sample to remove the shorter fragments and concentrate the DNA sample. The concentration of the sheared and purified DNA was assessed using a Nanodrop spectrophotometer and Qubit Fluorometer and Qubit dsDNA High Sensitivity Assay kit. Fragment size distribution was evaluated by running the sample on the FemtoPulse system.

### Sequencing

Pacific Biosciences HiFi circular consensus and 10X Genomics read cloud DNA sequencing libraries were constructed according to the manufacturers’ instructions. DNA sequencing was performed by the Scientific Operations core at the WSI on Pacific Biosciences SEQUEL II (HiFi) and HiSeq X Ten (10X) instruments. Hi-C data were also generated from tissue of ilAriAges1,ilAriAges2 using the Arima2 kit and sequenced on the HiSeq X Ten instrument.

### Genome assembly, curation and evaluation

For ilAriAges1, assembly was carried out with Hifiasm ( Cheng *et al.*, 2021), while for ilAriAges2, assembly was performed using Hicanu ( Nurk *et al.*, 2020). The same procedure was followed for the rest of the steps. Haplotypic duplication was identified and removed with purge_dups ( Guan *et al.*, 2020). One round of polishing was performed by aligning 10X Genomics read data to the assembly with Long Ranger ALIGN, calling variants with FreeBayes ( Garrison & Marth, 2012). The assembly was then scaffolded with Hi-C data ( Rao *et al.*, 2014) using SALSA2 ( Ghurye *et al.*, 2019). The assembly was checked for contamination and corrected using the gEVAL system ( Chow *et al.*, 2016) as described previously ( Howe *et al.*, 2021). Manual curation was performed using gEVAL, HiGlass ( Kerpedjiev *et al.*, 2018) and Pretext ( Harry, 2022). The mitochondrial genome was assembled using MitoHiFi ( Uliano-Silva *et al.*, 2022), which runs MitoFinder ( Allio *et al.*, 2020) or MITOS ( Bernt *et al.*, 2013) and uses these annotations to select the final mitochondrial contig and to ensure the general quality of the sequence.

A Hi-C map for the final assembly was produced using bwa-mem2 ( Vasimuddin *et al.*, 2019) in the Cooler file format ( Abdennur & Mirny, 2020). To assess the assembly metrics, the *k*-mer completeness and QV consensus quality values were calculated in Merqury ( Rhie *et al.*, 2020). This work was done using Nextflow ( Di Tommaso *et al.*, 2017) DSL2 pipelines “sanger-tol/readmapping” ( Surana *et al.*, 2023a) and “sanger-tol/genomenote” ( Surana *et al.*, 2023b). The genome was analysed within the BlobToolKit environment ( Challis *et al.*, 2020) and BUSCO scores ( Manni *et al.*, 2021; Simão *et al.*, 2015) were calculated.

Table 4 contains a list of relevant software tool versions and sources.

**Table 4.** Software tools: versions and sources.

Software tool	Version	Source
BlobToolKit	4.1.5	https://github.com/blobtoolkit/blobtoolkit
BUSCO	5.3.2	https://gitlab.com/ezlab/busco
FreeBayes	1.3.1-17-gaa2ace8	https://github.com/freebayes/freebayes
gEVAL	N/A	https://geval.org.uk/
Hicanu	2.1	https://github.com/marbl/canu
Hifiasm	0.12	https://github.com/chhylp123/hifiasm
HiGlass	1.11.6	https://github.com/higlass/higlass
Long Ranger ALIGN	2.2.2	https://support.10xgenomics.com/genome-exome/software/pipelines/latest/advanced/other-pipelines
Merqury	MerquryFK	https://github.com/thegenemyers/MERQURY.FK
MitoHiFi	1	https://github.com/marcelauliano/MitoHiFi
PretextView	0.2	https://github.com/wtsi-hpag/PretextView
purge_dups	1.2.3	https://github.com/dfguan/purge_dups
SALSA	2.2	https://github.com/salsa-rs/salsa
sanger-tol/genomenote	v1.0	https://github.com/sanger-tol/genomenote
sanger-tol/readmapping	1.1.0	https://github.com/sanger-tol/readmapping/tree/1.1.0

### Genome annotation

The Ensembl gene annotation system ( Aken *et al.*, 2016) was used to generate annotation for the *Aricia agestis* assemblies. Annotation was created primarily through alignment of transcriptomic data to the genome, with gap filling via protein-to-genome alignments of a select set of proteins from UniProt ( UniProt Consortium, 2019).

### Wellcome Sanger Institute – Legal and Governance

The materials that have contributed to this genome note have been supplied by a Tree of Life collaborator. The Wellcome Sanger Institute employs a process whereby due diligence is carried out proportionate to the nature of the materials themselves, and the circumstances under which they have been/are to be collected and provided for use. The purpose of this is to address and mitigate any potential legal and/or ethical implications of receipt and use of the materials as part of the research project, and to ensure that in doing so we align with best practice wherever possible. The overarching areas of consideration are:

•   Ethical review of provenance and sourcing of the material

•   Legality of collection, transfer and use (national and international)

Each transfer of samples is undertaken according to a Research Collaboration Agreement or Material Transfer Agreement entered into by the Tree of Life collaborator, Genome Research Limited (operating as the Wellcome Sanger Institute) and in some circumstances other Tree of Life collaborators.

## Data Availability

European Nucleotide Archive: *Aricia agestis* (brown argus). Accession number PRJEB42115; https://identifiers.org/ena.embl/PRJEB42115. ( Wellcome Sanger Institute, 2022) The genome sequence is released openly for reuse. The *Aricia agestis* genome sequencing initiative is part of the Darwin Tree of Life (DToL) project. All raw sequence data and the assembly have been deposited in INSDC databases. Raw data and assembly accession identifiers are reported in Table 1.

## Author information

Members of the Wellcome Sanger Institute Tree of Life programme are listed here: https://doi.org/10.5281/zenodo.4783585.

Members of Wellcome Sanger Institute Scientific Operations: DNA Pipelines collective are listed here: https://doi.org/10.5281/zenodo.4790455.

Members of the Tree of Life Core Informatics collective are listed here: https://doi.org/10.5281/zenodo.5013541.

Members of the Darwin Tree of Life Consortium are listed here: https://doi.org/10.5281/zenodo.4783558.
