# Supplementary material for: The genome sequence of the Brown Argus, Aricia agestis (Denis & Schiffermüller, 1775)
Source: Wellcome Open Res. 2023 Aug 10;8:336. [Version 1] doi: 10.12688/wellcomeopenres.19784.1 (PMC10884594; doi:10.12688/wellcomeopenres.19784.1)
Supplement: Peer review history

# Reviewer report: Reviewer response for version 1

**DOI:** 10.21956/wellcomeopenres.21912.r74513

**Published:** 2024-02-21

**Authors:** Benoit Nabholz

**Affiliations:** 1 Universite de Montpellier, Montpellier, Occitanie, France

The manuscript presents the genome of the Brown Argus ( *Aricia agestis*) produced by the Darwin Tree of Life project. This Polyommatinae species has an unusual chromosome number compared to the canonical 30 chromosomes of butterflies. The introduction mentions "white spots on the upper forewing" as a distinguishing characteristic from its sister species *A. artaxerxes*. However, I haven't found this feature in my reference literature  (Lafranchis, 2007 [Ref 1]).

 Unusually, two genomes of the same species are produced here. The genome assemblies are extremely similar in terms of size and number of annotated protein-coding genes, demonstrating the high quality of the pipeline used and of the data produced by the project. This is interesting, as high-quality genomes are rarely produced in the same species, and this could provide interesting insight into complex and large mutation polymorphisms (such as duplications, insertions, etc.). Once again, the project has all my support, and I congratulate for your work.

Are sufficient details of methods and materials provided to allow replication by others?

Yes

Is the rationale for creating the dataset(s) clearly described?

Yes

Are the datasets clearly presented in a useable and accessible format?

Yes

Are the protocols appropriate and is the work technically sound?

Yes

Reviewer Expertise:

Molecular evolution

I confirm that I have read this submission and believe that I have an appropriate level of expertise to confirm that it is of an acceptable scientific standard.

## References

[1] : Papillons d’Europe: guide et clés de détermination des papillons de jour. *Diatheo. Paris* .2010;

# Reviewer report: Reviewer response for version 1

**DOI:** 10.21956/wellcomeopenres.21912.r65073

**Published:** 2023-09-20

**Authors:** Xueyan Li

**Affiliations:** 1 State Key Laboratory of Genetic Resources and Evolution, Kunming Institute of Zoology, Chinese Academy of Sciences, Kunming, China

This manuscript provides two high-quality genome assemblies of two male specimens of the butterfly *Aricia agestis*, which is a very important genome resource of Lepidoptera, especially for further studies on host plant shifts associated with climate change, as well as ecological specialisation and speciation in the genus *Aricia*. Nevertheless, I have some questions as follows.

  

1. What is the aim to sequence and assemble the genomes of two individuals for the same butterfly species?
2. Please give some explanations for the differences of two assemblies (genome size, protein coding gene number).
3. Why were two different softwares selected to assemble two specimen genomes?

 

Are sufficient details of methods and materials provided to allow replication by others?

Yes

Is the rationale for creating the dataset(s) clearly described?

Yes

Are the datasets clearly presented in a useable and accessible format?

Yes

Are the protocols appropriate and is the work technically sound?

Yes

Reviewer Expertise:

Insect genome

I confirm that I have read this submission and believe that I have an appropriate level of expertise to confirm that it is of an acceptable scientific standard.
